# What a mesh: a call for better science and regulatory oversight

**DOI:** 10.1093/bjs/znag042

**Published:** 2026-05-07

**Authors:** Andrew W Kirkpatrick, Phillip M Millar, Christopher J Doig

**Affiliations:** TeleMentored Ultrasound Supported Medical Interventions (TMUSMI) Research Group, Regional Trauma Services, Calgary, Alberta, Canada; Department of Surgery, Cumming School of Medicine, University of Calgary, Calgary, Alberta, Canada; Department of Critical Care Medicine, Cumming School of Medicine, University of Calgary, Calgary, Alberta, Canada; Millars Law, London, Ontario, Canada; Department of Critical Care Medicine, Cumming School of Medicine, University of Calgary, Calgary, Alberta, Canada; Department of Community Health Sciences, Cumming School of Medicine, University of Calgary, Calgary, Alberta, Canada

## Editorial

Hernias have challenged humans since the earliest descriptions of medicine and impose a tremendous burden of disease. Hernias also illustrate great disparity in Global Health, with inordinate potentially preventable deaths in the developing world due to surgical unavailability. This is ironic, as repairing an inguinal hernia is more cost-effective on a health economy basis than many other recognized interventions^1^. Another complexity is that human (especially male) groin anatomy often has structural weakness such that when hernias reoccur there may be no reliable way to repair them with autologous tissue. The introduction of mesh-augmented hernia repairs permits surgeons to repair both simple and previously unrepairable hernias with improved results considering recurrence and disability. Hernia mesh is a medical device with a flat, flexible weave that supports tissue around a hernia as it heals. The most common hernia meshes are composed of polypropylene (PP). Therefore, mesh is an important if not obligate tool in the hernia surgeon’s armamentarium. However, hernia meshes have potential side effects including difficulty in obtaining microbiological cure without removal if infected, contraction and ‘meshoma’ formation, and erosion into vital organs if implanted in particular anatomical locations^2^. In the vast majority of patients, however, lives are improved through successful repair and a return to high function.

Recently, a new potential complication of mesh use in hernia repair has been postulated. The entity known as auto-inflammatory syndrome induced by adjuvants (ASIA)^3,4^ associates multiple non-specific visceral and somatic complaints with PP implantation. The syndrome has only been reported in methodologically limited uncontrolled case series using unsubstantiated diagnostic criteria^3^. To date, the least-worst scientific evidence refutes a relationship between PP mesh implantation and any specific autoimmune inflammatory syndrome, including ASIA^5–7^.

Unfortunately for patients seeking guidance to inform decision-making regarding their health, social media sources and legal enterprises have capitalized on poor science to create uncertainty and fear concerning injury from unsubstantiated mesh complications including potential autoimmune side effects. Three of the top five social media ‘Tweeters’ regarding hernia mesh and pelvic/vaginal mesh were linked to law firms involved in mesh-based lawsuits; further, hundreds of millions of dollars a year are directed to advertisements to recruit patients with concerns about being ‘mesh-injured’^8^. Thus, the current medicolegal cauldron surrounding surgical mesh is increasingly divorced from objective science. Fuelled by opaque third-party litigation funding, the mass tort industry has weaponized social media to manufacture litigation demand. By exploiting the scientific uncertainty surrounding postulated conditions, highly capitalized legal marketing networks systematically steer public sentiment to aggregate tens of thousands of claims. However, medical device regulation also shares culpability for this vulnerable environment. For decades, manufacturers have bypassed rigorous clinical trials by relying on regulatory loopholes, such as the US Food and Drug Administration’s 510(k) clearance pathway, which clears devices based merely on ‘substantial equivalence’ to archaic, pre-1976 predicate-devices. To rescue the patient–surgeon relationship from these competing, self-serving financial interests, we must fundamentally shift our standards.

It is into this charged, self-interest biased, and social media-influenced environment that Gielen and colleagues report the first study to date examining cohorts having surgery with implanted mesh to a surgical cohort without mesh regarding subsequent diagnosis of autoimmune disease (AID)^5^. Their findings are simple and straightforward: ‘mesh implants do not increase medium-term risk for developing AID’^5^. These conclusions are consistent with other recent meta-analyses that have concluded that ‘there is little evidence that the use of polypropylene mesh can lead to autoimmunity’^7^, and as worded by Kowalik and colleagues, ‘there is no evidence to suggest a causal relationship between being implanted with a PP mesh and the occurrence of autoimmune disorders’^6^. Gielen and colleagues further hope that their evidence might reduce litigation related to hernia mesh^5^.

This helps surgeons to better discuss risks and benefits with patients and the decision whether to use a PP mesh in the repair. It can be accepted that the risk of hernia recurrence will be reduced at the small risk of a local mesh complication. Local mesh complications can also be reduced through surgical experience in selecting the correct anatomical location to place the mesh. Additionally, as emphasized by both Kowalik and Gielen and colleagues, surgeons should be comfortable in counselling patients regarding the benefits of a mesh-augmented hernia repair. As well, surgeons can communicate that based on current evidence there is no basis to remove previously implanted PP mesh in order to resolve perceived autoimmune complications^5,6^.

On reflection, it is a condemnation of contemporary medical science that we do not have clear unequivocal advice for our patients on how best to perform a procedure preformed 20 million times a year. Most reviews on this topic conclude that better scientific evidence is required. To rescue the patient–surgeon relationship from these competing, self-serving financial interests, we must fundamentally shift our standards. Mandatory, prospective clinical registries and independent expert panels are urgently required to establish irrefutable clinical evidence, permanently replacing the linear, financially motivated narratives. It thus seems logical that every procedure performed is entered into a prospective registry, and that every surgeon practising within a university setting be required to participate in randomized trials, and every patient whose care is paid for by a publicly funded healthcare system should be expected to participate in ethically approved studies to identify how to best care for the entire population. Apart from being scientifically and ethically valid, the requirement to participate has a moral imperative as described by Schaefer^9^. Alas, this is far from reality, and medical devices such as implantable mesh do not even typically need research to be marketed in North America. Thus, until the entire surgical community and patients’ advocates for universally better science and a fundamental societal shift in our *pro tanto* moral obligations to society^9,10^, the evidence and thoughtful interpretation of Gielen’s and colleagues should be greatly appreciated by clinicians and patients.
